# Proteomic characteristics of saliva in patients with different subgroups of IgG4-RD

**DOI:** 10.3389/fimmu.2022.1026921

**Published:** 2022-11-22

**Authors:** Sheng-Yan Lin, Tianshu Zhou, Shaozhe Cai, Zi-Wei Hu, Jixin Zhong, Lingli Dong

**Affiliations:** Department of Rheumatology and Immunology, Tongji Hospital, Tongji Medical College, Huazhong University of Science and Technology, Wuhan, China

**Keywords:** IgG4-related disease (IgG4-RD), saliva, proteomic analysis, IgG4-RD subgroups, IgG 4-RD pathogenesis

## Abstract

**Background:**

Immunoglobulin G4-related disease (IgG4-RD) is a newly defined disease entity, with great heterogeneity among IgG4-RD subgroups with different organ involvement patterns. Identification of the proteomic characteristics of IgG4-RD subgroups will be critical for the understanding of the pathogenic mechanisms of IgG4-RD.

**Method:**

In this study, we performed proteomic analysis using Tandem Mass Tags (TMT) technology with “high field” mass analyzer with improved resolution and sequencing speed to investigate the proteomic profile of saliva and plasma samples from ten untreated IgG4-RD patients and five healthy controls (HCs). Differentially expressed proteins (DEPs) were identified by “t test” function in R package. Functional enrichment analysis was used to investigate pathways enriched in IgG4-RD samples.

**Results:**

Most salivary DEPs identified in IgG4-RD patients compared with HCs were mainly enriched in neutrophil mediated GO bioprocess. Within the comparisons between four IgG4-RD subgroups, more DEPs were identified in the comparison of Mikulicz group and Head and neck group. Among four subgroups of IgG4-RD, Head and neck group showed the most distinctive proteomic expression pattern when compared with HCs. Moreover, “Neutrophil mediated process” related GO bioprocess was commonly identified between comparisons of Mikulicz group and Head and neck group, Head and neck group and Retroperitoneal aorta group, Head and neck group and HCs, IgG4-RD patients with saliva gland involvement and those without saliva gland involvement. Key DEPs that involved in this GO bioprocess were identified. Besides, we performed proteomic analysis for plasma samples between ten IgG4-RD and five HCs and there were several DEPs identified overlapped in saliva and plasma.

**Conclusion:**

We identified multiple processes/factors and several signaling pathways in saliva that may be involved in the IgG4-RD pathogenesis.

## Introduction

Immunoglobulin G4-related disease (IgG4-RD) is a recently recognized immune-mediated disease that was characterized by multiple organ involvements, elevated serum IgG4 levels, and an infiltration of polyclonal IgG4-positive (IgG4^+^) plasma cell (1). Most affected organs include saliva glands, lacrimal glands, pancreas, paranasal sinus, lung, and kidney (2). In general, IgG4-RD affects more men than women and age at diagnosis ranges from 50 to 70 years (3). Although most IgG4-RD patients respond well to glucocorticoid treatment, the relapse rate is nearly 50% (4).

At present, IgG4-RD poses significant challenges in etiological understanding, in part because of their heterogeneous manifestations. Since its first description in 2003, the recognition of the multifaceted presentations of IgG4-RD has gradually improved (5). In 2012, a Japanese research team published the first diagnostic criteria of IgG4-RD, so that the diagnosis of IgG4-RD could be standardized (6). In 2019, Wallace et al. performed a latent class analysis to the largest international cohort of IgG4-RD patients and characterized four distinct phenotypes based on organ involvement patterns, including pancreato-hepato-biliary disease, retroperitoneal fibrosis and/or aortitis, head and neck-limited disease, and classic Mikulicz**’**s syndrome with systemic involvement (7). Then, Lanzillotta et al. confirmed the reproducibility of this classification in clinical practice and its relevance for IgG4-RD patient management (8). These investigations may serve as a framework for further identifying IgG4-RD and providing optimal care to patients. However, despite progress in the diagnosis and treatment of IgG4-RD, the exact proteomic characteristics pathogenic mechanism of four distinct phenotypes of IgG4-RD remains unclear.

In recent years, researchers applied RNA-seq or DNA microarray analysis to discover biomarkers and provided preliminary information on the underlying pathogenesis of IgG4-RD (9 (9),. Since disease states usually involve alterations in the post-transcriptional modifications of proteins, proteomics techniques have also been applied in rare diseases to search for biological information that could further our understanding of pathophysiological mechanisms and identify novel disease biomarkers (10). For example, mass spectrometry (MS) based quantitative proteomics techniques were applied to obtain accurate and comprehensive protein profile of small samples in rare disease (10). In our previous work, we detected both serologic and tissue proteasome between IgG4-RD patients and healthy controls and identified multiple processes/factors and several signaling pathways involved in IgG4-RD (11). However, those studies did not elucidate the differences between four distinct IgG4-RD phenotypes and the key molecules are unclear.

In the past decade, salivary proteome analysis has gradually evolved in different biomedical fields such as genetics, molecular biology, and medicine (12). Several studies have reported that saliva may be applied as a diagnostic fluid to detect oral diseases such as periodontitis, oral squamous cell carcinoma, and Sjögren’s syndrome (13). Moreover, the saliva proteome has already been used to evaluate its change in immune-mediated inflammatory diseases including diabetes mellitus (14), cystic fibrosis (15), and multiple sclerosis (16), revealing the great potential of proteomics in biomarker identification and providing new insight into the molecular mechanisms underlying inflammatory diseases. Moreover, the collection of saliva samples is usually easy to perform, economical, and safe. Considering the saliva gland is one of the most commonly involved sites of IgG4-RD (17) and saliva may become altered in response to various diseases (18), we collected saliva samples to study the proteasome of four subtypes of IgG4-RD.

In this paper, through comprehensive proteomic analyses, we identified several differently expressed proteins in saliva samples between IgG4-RD patients and healthy controls. In addition, we analyzed the difference of expression and pathways among IgG4-RD patients with four distinct phenotypes. Our work provides insights to the molecular difference of different IgG4-RD phenotypes and will be useful to IgG4-RD classification and prognosis prediction.

## Materials and methods

### Clinical patient information

In this research, we enrolled ten newly diagnosed treatment-naïve patients IgG4-RD patients, and five sex- and age-matched healthy controls (HCs). All IgG4-RD patients who fulfilled the 2011 comprehensive IgG4-RD diagnostic criteria (6) were recruited in the Department of Rheumatology and Immunology, Wuhan Tongji Hospital (China) between July 2020 and January 2021. Patients with other autoimmune diseases, active/severe infection and malignant diseases were excluded. Based on organ involvement patterns (7), ten IgG4-RD patients were further divided into four subgroups: A. classic Mikulicz’s syndrome with systemic involvement (n = 3), named as Mikulicz group; B. Pancreato-hepato-biliary disease (n = 2), named as Hepatobiliary group; C. Head and neck-limited disease (n = 2), named as Head and neck group, and D. Retroperitoneal fibrosis and/or aortitis (n = 3), named as Retroperitoneal aorta group. Details of the patients’ clinical characteristics at baseline were shown in **Table 1**. As for controls, sex- and age-matched HCs who lacked any diagnosed chronic medical diseases and did not take prescription or over-the-counter medications within the past seven days were recruited from the community. All patients and HCs provided written informed consent to participate. Saliva and plasma samples from ten patients and five HCs were used for proteomics analysis.

**Table 1:** Main clinical characteristics of IgG4-RD patients in this study.

Group	Classification	Sex	Age	Disease duration (months)	Involved organ	IgG4/IgG ratio of plasma cells (Immunohistochemistry)	IgGlevel	IgG1level	IgG2level	IgG3level	IgG4level	IgElevel
A	Mikulicz group	Male	54	2	Submandibular gland	65%	11.26	5.78	5.93	0.34	2.97	101.3
A	Mikulicz group	Female	41	7	Submandibular gland	50%	9.1	/	/	/	1.45	16.1
A	Mikulicz group	Male	49	12	Submandibular gland	50%	13	7.17	5.54	0.562	0.539	43.88
B	Hepatobiliary group	Male	64	0	Biliary tract, liver	70%	26.22	12.5	2.02	0.655	16.6	533.4
B	Hepatobiliary group	Male	39	24	Biliary tract, Pancreas	50%	21.3	13.6	4.25	0.664	9.58	414.1
C	Head and neck group	Female	71	1	Vocal cord, thyroid	40%	20.32	12.5	5.75	0.64	1.76	/
C	Head and neck group	Female	31	24	Parotid gland, lacrimal gland	70%	19.7	11.4	5.53	0.853	10.4	1127
D	Retroperitoneal fibrosis group	Male	56	2	Retroperitoneum, Kidney, aorta	70%	16.71	10.9	7.29	0.787	1.65	63.12
D	Retroperitoneal fibrosis group	Male	37	1	Aorta, kidney	/	22.42	16.3	/	/	4.1	/
D	Retroperitoneal fibrosis group	Male	76	1	Retroperitoneum, lung	/	12.47	7.76	4.76	0.357	2.43	304.9

Symbol “/” means information is missing.

### Sample collection

Saliva was collected between 8–10 a.m. All enrolled IgG4-RD patients and HCs were refrained from eating, drinking, and oral hygiene procedures for at least 1 h before saliva collection. Before collection, a 1 min oral rinse with distilled water was performed and ~5 mL of saliva was then collected in the next 5 min as described (19). The collected samples were processed in the laboratory with sterilized vessels within 1 h and stored at −80°C for further analysis. Meanwhile, whole blood of enrolled IgG4-RD patients and HCs was harvested by venipuncture into EDTA‐containing sampling containers. The blood was centrifuged at 4000rpm for 5 min, and the supernatant was stored at −80°C for further proteomics analysis.

### Protein extraction, processing, and MS/MS analysis

In this study, the Tandem Mass Tags (TMT) technology was applied to investigate the proteasome of saliva and plasma samples (SpecAlly Life Technology Co., Ltd, Wuhan, China). For saliva samples, fraction supernatants were loaded on an UHPLC system (Thermo Scientific™ UltiMate™ 3000) equipped with a trap and an analytical column, and peptides separated from nanoHPLC were subjected into the tandem mass spectrometry Orbitrap Exploris 480 (Thermo Fisher Scientific, San Jose, CA) for DDA detection (20). Scans were carried out with the following parameters: the ion source voltage: 1.6 kV; precursor scan range: 350-1800 m/z; MS/MS fragment scan range: >100 m/z at a resolution of 35,000 in HCD mode; HCD collision energy setting: 32; dynamic Exclusion time: 35s; automatic gain control (AGC) for full MS target and MS2 target: 3e6 and 2e5, respectively; the number of MS/MS scans following one MS scan: 15.

For plasma samples, the ProteoMiner Protein Enrichment Kit (Bio-rad laboratories, Hercules, CA, USA) was applied to deplete the high abundance proteins. Then, trypsin or Glu-C digestion was performed on two parallel batches of the samples. The resulting peptides were separated and detected using a Q-Exactive tandem mass spectrometer equipped with an Orbitrap analyzer (Thermo Fisher Scientific) (21). In brief, mixed peptides were obtained by using a C18-Gemini column (100μm×20mm, 120 Å, 3 μm particle size) and subsequently fractioned by separation column (750μm×150mm, 120 Å, 2 μm particle size) on the EASY-nLC 1200 system. Finally, fraction supernatants were loaded on UHPLC system (Thermo Scientific™ UltiMate™ 3000) equipped with a trap and an analytical column, and peptides separated from nanoHPLC were subjected into the tandem mass spectrometry Orbitrap Exploris 480 (Thermo Fisher Scientific, San Jose, CA) for data-dependent acquisition (DDA) detection by nano-electrospray ionization. Scans were carried out with the following parameters: the ion source voltage: 1.6 kV; precursor scan range: 350-1800 m/z; MS/MS fragment scan range: >100 m/z at a resolution of 15,000 in higher-energy collisional dissociation (HCD) mode; HCD collision energy setting: 30; dynamic Exclusion time: 35s; automatic gain control (AGC) for full MS target and MS2 target: 300% and 100%, respectively; the number of MS/MS scans following one MS scan: 20.

### Protein identification and quantification

Protein identification and quantification were realized by software MaxQuant (22). The mass spectra data was retrieved by MaxQuant v1.6.6 and the retrieval algorithm was Andromeda in Protein knowledgebase (UniProtKB). The propensity score matchings (PSMs) were pre-filtered with false discovery rate (FDR) <=1% to assess the confidence of peptides and the anti-library protein, contaminating protein, and protein entries with only one modified peptide were deleted, and the remaining identification information was used for subsequent analysis. The raw mass spectrometry proteomics data of saliva and plasma samples from ten IgG4-RD patients and five healthy controls (HCs) have been deposited in the ProteomeXchange Consortium *via* the iProX partner repository (23) with the dataset identifier PXD036552 (http://proteomecentral.proteomexchange.org/cgi/GetDataset?ID=PXD036552).

### Differential expression analysis and data analysis

To identify the differentially expressed proteins (DEPs) between the IgG4-RD subgroups with different organ involvement pattern, we used ‘t test’ function in R package (version 3.6). The thresholds for differential analysis of DEGs were FDR ≤ 0.05 and |Ratio| >1.2. Functional enrichment annotations for GO and KEGG pathways were analyzed using the Database for Annotation, Visualization and Integrated Discovery online tool (https://david.ncifcrf.gov/). Pathways and GO terms with p ≤ 0.05 based on Fisher’s Exact Test were considered as statistically significant. Protein expression patterns were indicated by heatmaps, which were performed using R packages such as “gglot2” and “Complex Heatmap”. The immunologically relevant list of genes curated with functions (24) updated in July 2020 was downloaded from the ImmPort database (https://www.immport.org/shared/genelists). The STRING database (https://string-db.org/) was used to analyze the protein-protein interaction (PPI) network.

### Validation dataset

IgG4-RD microarray datasets GSE66465 (25) and GSE40568 (26) were collected from Gene Expression Omnibus (GEO) for validation. Specifically, peripheral blood mononuclear cell (PBMC) samples of IgG4-RD in datasets GSE66465 were obtained from patients with IgG4-RD before steroid therapy (n = 2) who registered in the research project of the Research Program for Intractable Disease of the Ministry of Health, Labor, and Welfare (MHLW) of Japan and HCs (n = 4). While in GSE40568, labial salivary glands (LSGs) samples of Sjögren’s syndrome (n = 5) and HCs (n = 3) were obtained. For each cohort, we performed differential expression analysis with the thresholds FDR < 0.05 and |Fold change| > 1.5 between the IgG4-RD and HCs groups using R survival package “t-test”.

## Results

### Identification of differentially expressed proteins in saliva between IgG4-RD patients and HC samples

**Figure 1:**
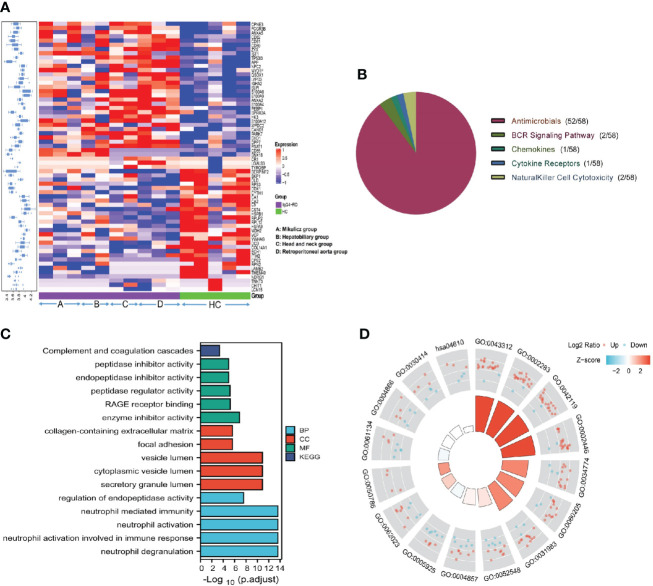
Summary of the DEPs in IgG4-RD and HC group in saliva. **(A)** The expression heatmap of DEPs in IgG4-RD and HC group in saliva. The blue to red in the right indicates the expression level from low to high. Proteins have carried out hierarchical clustering by calculating average distances. **(B)** Immune categories of DEPs identified in saliva between IgG4-RD patients and HC samples. Different color represents various immune related categories from IMMPORT database. **(C)** Enriched GO terms of differentially expressed proteins in saliva between IgG4-RD patients and HC groups. **(D)** Each column in the inner ring corresponds to a GO category, and the height of each column represents the p.adjust value (higher the column, smaller the p.adjust value). The color of each GO category represents the Z-score value, and “up” and “down” label represents the molecule category based on Log2 Ratio calculation, respectively.

Totally, 64 (35 up-regulated, 29 down-regulated) DEPs in saliva (**Figure 1A**) were identified and the top10 upregulated DEPs (rank by ratio) in IgG4-RD were SLPI, S100A12, S100A8, S100A9, CD55, LYPD3, CD59, IGHA2, APP and ANXA5. Among them, the S100 family, including S100A12, S100A8 and S100A9, may act as calcium- and zinc-binding protein and play prominent roles in the regulation in the recruitment of leukocytes, promotion of cytokine and chemokine production (27). By using IMMPORT database, most salivary DEPs (52/58), including CD55, CD59, IGHA2, APP and ANXA5, mainly belong to antimicrobials category (**Figure 1B** and **Table S1**). To understand the function of these DEPs in IgG4-RD, functional enrichment analysis was performed. Results indicated that most saliva DEPs in IgG4-RD patients were involved in neutrophil mediated GO Bioprocess including neutrophil degranulation (GO:0043312), neutrophil activation involved in immune response (GO:0002283), neutrophil activation (GO:0042119) and neutrophil mediated immunity (GO:0002446) (**Figures 1C, D**). We analyzed correlations between expression of those DEPs and serum immunoglobulin levels including IgG, IgG1, IgG2, IgG3, IgG4, and toal IgE. In saliva, PARK7 expression was positively correlated with IgG, IgG1, and IgG4 levels, while CST4 expression was negatively correlated with IgG, IgG1, and IgG4 levels (**Table 2**).

**Table 2:** Differentially expressed proteins identified in saliva correlated with serum immunoglobulin level of IgG4-RD patients (Rank by correlation value).

Biological factor	Protein	Correlation	p-value	up/down in IgG4-RD
IgG	PARK7	0.94	0.0017	up
IgG1	PARK7	0.92	0.0035	up
IgG4	PARK7	0.89	0.0077	up
IgG1	CA1	0.88	0.0097	down
IgG4	TP53I3	0.84	0.0191	up
IgG	CPB2	0.81	0.0282	down
IgG1	PEBP1	0.81	0.0283	up
Total IgE	TP53I3	0.78	0.0367	up
IgG4	CST4	-0.75	0.0501	down
IgG1	CST4	-0.78	0.0366	down
IgG2	TP53I3	-0.79	0.0343	up
IgG2	CPB2	-0.79	0.0329	down
Total IgE	DCD	-0.82	0.0236	down
IgG	CST4	-0.83	0.0204	down

### Identification of differentially expressed proteins among different IgG4-RD subgroups

**Figure 2:**
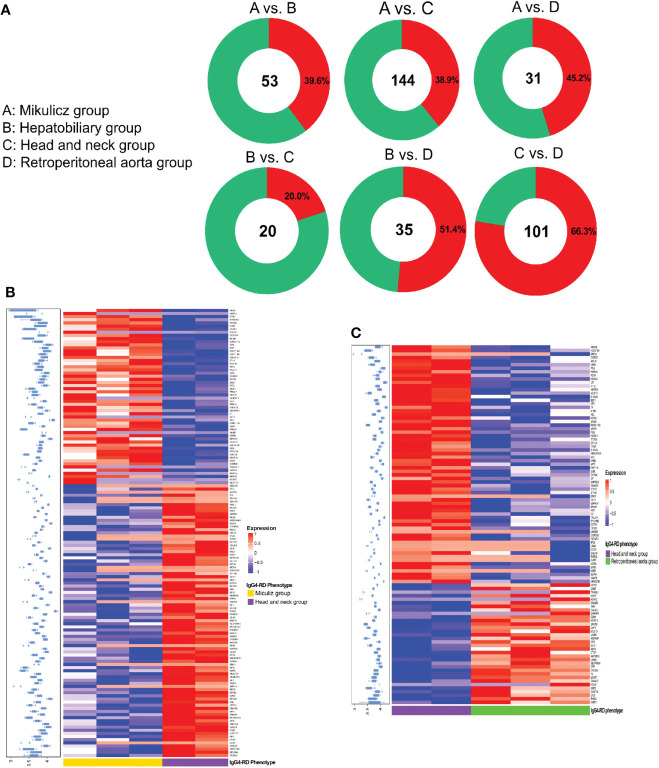
Summary of DEPs among IgG4-RD patients of distinctive phenotypes in saliva. **(A)** The number and ratio for up/down-expressed DEPs in comparisons of A vs. B, A vs. C, A vs. D, B vs. C, B vs. D and C vs. D in IgG4-RD in saliva. Note: A: Mikulicz group; B: Hepatobiliary group; C: Head and neck group; and D: Retroperitoneal aorta group. Red and green circle represents up and down expressed DEPs, respectively. **(B)** The expression heatmap of DEPs in IgG4-RD of Mikulicz group and Head and neck group. **(C)** The expression heatmap of DEPs in IgG4-RD of Head and neck group and Retroperitoneal aorta group.

We performed differential protein analysis among different IgG4-RD subgroups: (A) Mikulicz group, (B) Hepatobiliary group, (C) Head and neck group and (D) Retroperitoneal aorta group, and we obtained a list of DEPs from each comparison. In general, there were much more DEPs identified in the comparisons of Mikulicz group or Retroperitoneal aorta group versus Head and neck group than in other comparisons (**Figure 2A**) and heat maps of DEPs in those comparisons were shown in **Figures 2B, C**. Among them, IGLV2-11 and BPGM were the overlapped up- and down-regulated DEPs in the comparisons of Mikulitz group versus Hepatobiliary group, Head and neck group, and Retroperitoneal aorta group, respectively. FGB was upregulated in the Mikulitz Group, Hepatobiliary group, and Head and neck group compared with the Retroperitoneal aorta group (marked in Red). UGDH was upregulated in Hepatobiliary group compared with Mikulicz group, Head and neck group and Retroperitoneal aorta group. In addition, ACAP2 and CRNN were found upregulated in the Mikulicz group, Hepatobiliary group, and Retroperitoneal aorta group compared with Head and neck group, while FCN1 was downregulated (**Table 3**). Interestingly, the ACAP2 expression was also decreased in Head and neck group compared with HCs (**Table S3**).

**Table 3:** Common up and down DEPs in saliva between four IgG4-RD subgroups.

Compared group	Up expressed	Compared group	Down expressed
A/B A/C A/D	IGLV2-11	A/B A/C A/D	BPGM
A/D B/D C/D	FGB	A/B A/D C/D	HSPE1
B/A B/C B/D	UGDH	/	/
A/C B/C D/C	ACAP2 CRNN	A/C B/C D/C	FCN1
A/B A/C	PODXL COL6A2 VS. IG10L	A/B A/C	FH SEPTIN2 DPP3 PARK7 PGM3 TFG BAG3
A/C A/D	IGLV7-43 LSR DAG1	A/C A/D	PSMA4 ALDH16A1 MARK2 NUDC HNRNPL HSPA8
A/C B/C	ACAP2 CRNN	A/C B/C	MAGOH8 RETN FCN1 NIT2 HACD3
A/D B/D	FGG	A/D B/D	MOGS
B/D C/D	VAMP8 GALNS TYMP TP53I3 MAPRE2 MPST	B/D C/D	NADK HSPA13 LGMN
B/C B/D	HSPE1 UGDH	A/B A/D	PGM2 PTGR1
B/C C/D	DCXR LSM4 GATD1	A/D C/D	CDH1 UAP1

A. Mikulicz group, B. Hepatobiliary group, C. Head and neck group, D. Retroperitoneal aorta group. Symbol “/” means no common DEPs identified in compared group.

**Figure 3:**
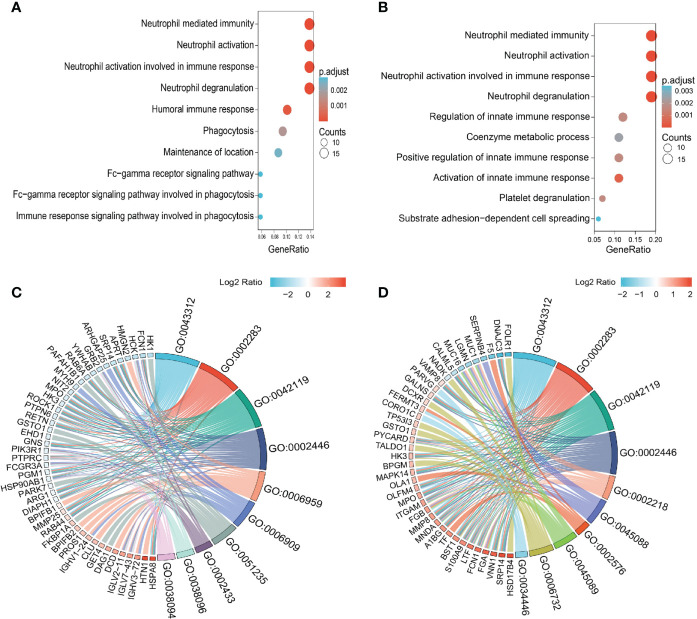
Enriched GO categories of DEPs in the comparison between IgG4-RD with four subgroups in saliva. **(A)** Enriched GO categories of DEPs in the comparison between Mikulicz group vs. Retroperitoneal aorta group. **(B)** Enriched GO categories of DEPs in the comparison between Head and neck group vs. Retroperitoneal aorta group. **(C)** Chordal diagram of GO categories in comparison between Mikulicz group vs. Retroperitoneal aorta group. **(D)** Chordal diagram of GO categories in the comparison between Head and neck group vs. Retroperitoneal aorta group. The left half part is the gene color block and different color represents the corresponding gene Log2 Ratio values, the right half is the GO category color block, and the size of the color block represents the corresponding counts.

Knowledge of IgG4-RD in head and neck regions is important, since the salivary gland is one of the most frequently involved sites (28). In this paper, we analyzed all DEPs in the comparisons of Mikulicz group with Head and neck group (A vs. C), and Head and neck group with Retroperitoneal aorta group (C vs. D) for functional enrichment. We found that “Neutrophil mediated process” related GO BP term that includes neutrophil degranulation (GO:0043312), neutrophil activation involved in immune response (GO:0002283), neutrophil activation (GO:0042119) and neutrophil mediated immunity (GO:0002446) was identified in both comparisons (**Figures 3A, B**), which both include FCN1, MPO and HK3 (**Figures 3C, D**) and all these proteins were increased in Head and neck group compared with Mikulicz group or Retroperitoneal aorta group (**Table S2**). In addition, our study identified a significant enrichment of “Fc gamma-mediated phagocytosis” process in Mikulicz group compared with Head and neck group in saliva (**Figure 3A**). Notably, IGLV2-11 and IGLV7-43 were involved in the Fc-gamma receptor-signaling pathway and were increased in Mikulicz group compared with Head and neck group in saliva (**Figure 3C**). Interestingly, “Fc-gamma receptor signaling pathway” was also detected in IgG4-RD tissue in our previous paper (11), which may suggest a potential role in the pathogenesis of IgG4-RD Mikulicz group.

### Identification of differentially expressed proteins between IgG4-RD subgroup and HC saliva

**Figure 4:**
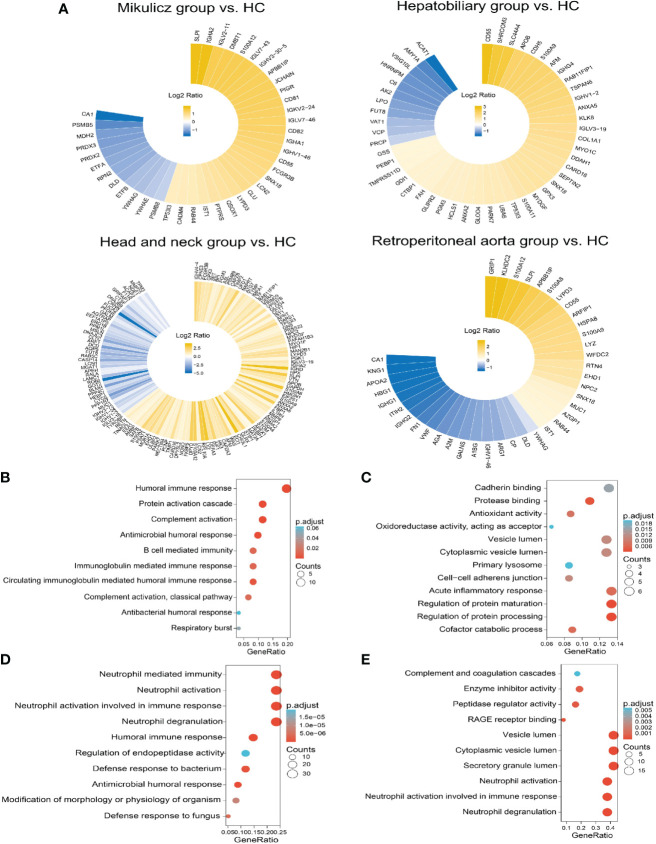
Summary of DEPs and enriched GO categories in the comparison between IgG4-RD with four subgroups and HC. **(A)** Circular heat map of up and down expressed of DEPs in comparisons of Mikulicz group vs. HC, Hepatobiliary group vs. HC, Head and neck group vs. HC and **(D)** Retroperitoneal aorta group vs. HC in saliva. Yellow and blue block represents the unexpressed and down expressed DEPs in each IgG4 phenotype, respectively. **(B)** Enriched GO category of DEPs in the comparison between Mikulicz group and HC. **(C)** Enriched GO category of DEPs in the comparison between Hepatobiliary group and HC. **(D)** Enriched GO category of DEPs in the comparison between Head and neck group and HC. **(E)** Enriched GO category of DEPs in the comparison between Retroperitoneal aorta group and HC.

**Figure 5:**
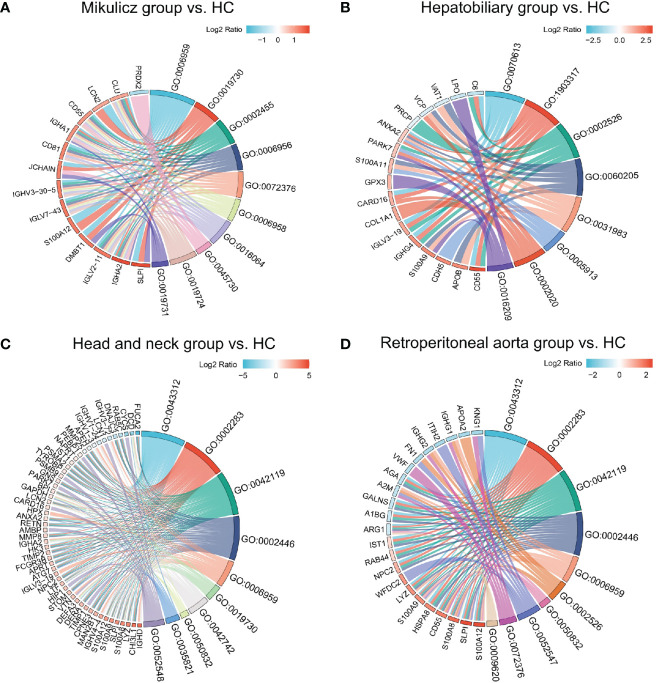
Chordal diagram of top ten GO categories in comparisons of IgG4-RD with four subgroups vs. HC. Chordal diagram of top ten GO categories in comparisons of **(A)** Mikulicz group vs. HC, **(B)** Hepatobiliary group vs. HC, **(C)** Head and neck group vs. HC, and **(D)** Retroperitoneal aorta group vs. HC in saliva. The left half part is the gene color block and different color represents the corresponding gene Log2 Ratio values, the right half is the GO category color block, and the size of the color block represents the corresponding counts.

Aiming to explore proteomic pattern of IgG4-RD saliva, we performed differential protein analysis between IgG4-RD subgroup and HC samples and obtained a list of DEPs from each comparison in saliva (**Table S3**). Overall, there were more DEPs identified in the comparison between Head and neck group and HC than other comparisons (**Figure 4A**). The overlapped DEPs in the comparison between IgG4-RD with four subgroups and HC were list in **Table 4**. Among them, TP53I3 was upregulated in the Mikulicz group, Hepatobiliary group, and Head and neck group compared with HC. CD55 and SNX18 were both upregulated in the Mikulicz group, Hepatobiliary group, and Retroperitoneal aorta group compared with HC. S100A12, IST1, LYPD3, and SLPI were upregulated in Mikulicz group, Head and neck group, and Retroperitoneal aorta group compared with HC. S100A9 was upregulated in Hepatobiliary group, Head and neck group, and Retroperitoneal aorta group (**Table 4**). We analyzed all DEPs from saliva in the comparisons between each IgG4-RD subgroup and HC respectively for functional enrichment, and top10 GO BP categories were shown in **Figure 4**. As a result, “Neutrophil mediated process” related GO BP term that includes neutrophil degranulation and neutrophil activation was found in the comparisons of Head and neck group/Retroperitoneal aorta group with HC in saliva (**Figures 4D, E**). CD55 was the key molecule involved in humoral immune related response (GO:0006959, GO:0002455 and GO:0016064) and inflammatory response related bioprocess (GO:0002526 and GO:0002673) in the comparisons of Mikulicz group /Hepatobiliary group with HC in saliva (**Figures 5A, B**). Moreover, LYZ, S100A8, S100A9, S100A12, IST1, and NPC2 were the key molecules involved in neutrophil mediated process (GO:0043312, GO:0002283, GO:0042119 and GO:0002446) and were increased in IgG4-RD compared with HC samples (**Figures 5C, D**).

**Table 4:** The overlapped DEPs in the comparison between IgG4-RD with four subgroups and HC.

Compared group	Up expressed	Down expressed
A_HC B_HC C_HC	TP53I3	/
A_HC B_HC D_HC	CD55 SNX18	/
A_HC C_HC D_HC	S100A12 IST1 LYPD3 SLPI	/
B_HC C_HC D_HC	S100A9	/
A_HC C_HC	LCN2 FCGR3B IGHA2	/
A_HC D_HC	RAB44 APBB1IP	YWHAG CA1 DLD
B_HC C_HC	GLOD4 ANXA2 AFM PARK7 APOB PGM3 IGLV3-19 MYDGF PEBP1 GPX3 CARD16 RAB11FIP1	/
C_HC D_HC	AZGP1 EHD1 S100A8 LYZ NPC2	/

A. Mikulicz group, B. Hepatobiliary group, C. Head and neck group, D. Retroperitoneal aorta group. “/” means no down expressed DEPs overlapped.

### Differences of saliva proteins between IgG4-RD patients with salivary gland involvement and without salivary gland involvement

**Figure 6:**
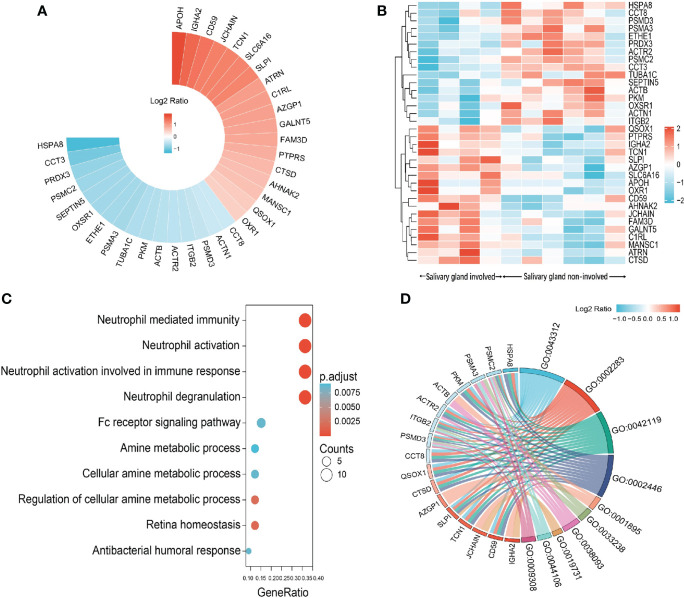
Summary of DEPs in the comparison between IgG4-RD patients with salivary gland involvement and without salivary gland involvement. **(A)** Circular heat map of upregulated and downregulated DEPs in the comparison between IgG4-RD patients with salivary gland involvement and without salivary gland involvement. **(B)** The expression heatmap of DEPs in IgG4-RD patients with salivary gland involvement and without salivary gland involvement. **(C)** Enriched GO category of DEPs in comparison of IgG4-RD patients with salivary gland involvement and without salivary gland involvement. **(D)** Chordal diagram of top ten GO categories in comparison of IgG4-RD patients with salivary gland involvement and without salivary gland involvement. The left half part is the gene color block and different color represents the corresponding gene Log2 Ratio values, the right half is the GO category color block, and the size of the color block represents the corresponding counts.

Saliva gland was reported as one of the most commonly involved sites of IgG4-RD (17), so we next compared the proteomic profiles between IgG4-RD patients with and without saliva gland involvement. Totally, 34 (18 up-regulated, 16 down-regulated) DEPs in saliva were identified between IgG4-RD patients with and without saliva gland involvement (**Figures 6A, B**). Functional enrichment analysis showed that DEPs were mainly enriched in “Neutrophil mediated related GO BP categories” including Neutrophil mediated immunity, Neutrophil activation, and Neutrophil degranulation terms (p<0.05) (**Figure 6C**) and DEPs in IgG4-RD patients with saliva gland involvement include CD59, CTSD, QSOX1, SLPI, TCN1, ACTR2, CCT8, HSPA8, ITGB2, PKM, PSMC2, and PSMD3 (**Figure 6D**). Among them, CD59, CTSD, QSOX1, SLPI, TCN1, and ACTR2 were upregulated, while CCT8, HSPA8, ITGB2, PKM, PSMC2, and PSMD3 were downregulated. We also examined correlations between DEPs and serum immunoglobulin levels including IgG, IgG1, IgG2, IgG3, IgG4, and total IgE. The expression of SLPI in saliva was positively correlated with IgG4, while HSPA8 was negatively correlated with IgG2 (**Table 5**), and HSPA8 decreased the most in IgG4-RD patients with saliva gland involvement in this study.

**Table 5:** Differentially expressed proteins correlated with important clinical parameters of IgG4-RD patients with salivary gland involvement in saliva.

Biological factor	Protein	Correlation	p-value	Up/down expressed in IgG4-RD
IgG3	PSMC2	0.984143	0.015857	down
IgG4	PSMA3	0.98284	0.01716	down
IgG4	PSMD3	0.972299	0.027701	down
IgG4	SLPI	0.964107	0.035893	up
IgG1	TUBA1C	0.962768	0.037232	down
IgG1	ETHE1	0.948682	0.051318	down
IgG	TUBA1C	0.947323	0.052677	down
IgG2	HSPA8	-0.94923	0.050775	down

(Rank by correlation).

### Differentially expressed proteins in the saliva and plasma of IgG4-RD and HC

In this study, we also collected plasma samples of ten IgG4-RD and five HC for analysis.

There are more DEPs detected in plasma than those in saliva (295 up-regulated and 188 down-regulated) (**Table S4**). Meanwhile, we found nine overlapped DEPs in the saliva and plasma (**Table 6**). Among them, three upregulated DEPs (SLPI, QSOX1, and LGALS3) and one downregulated DEP (VCP) showed the same expression tendency. LGALS3, which also known as galectin-3, is an autoantigen found in the plasma of a subset of IgG4-RD patients (29) and was increased in saliva of IgG4-RD patients in this study.

**Table 6:** Overlapped proteins identified in saliva and plasma of IgG4-RD patients.

Saliva				Plasma			
Protein	Ratio	p-value	Up/down	Protein	Ratio	p-value	Up/down
**SLPI**	2.35	0.0006	up	**SLPI**	0.78	0.0012	up
EHD1	0.89	0.0086	up	C5	0.61	0.0037	up
ZYX	0.77	0.0420	up	SERPINF2	0.55	0.0019	up
**QSOX1**	0.67	0.0092	up	**QSOX1**	0.50	0.0007	up
**LGALS3**	0.59	0.0110	up	**LGALS3**	0.44	0.0085	up
**VCP**	-0.41	0.0430	down	ITIH2	0.36	0.0194	up
C5	-0.60	0.0263	down	ZYX	-0.43	5.34E-05	down
ITIH2	-0.82	0.0456	down	**VCP**	-0.60	0.0030	down
SERPINF2	-1.69	0.0423	down	EHD1	-0.76	0.0020	down

Common upregulated and downregulated DEPs with same expression tendencies identified in the saliva and plasma of four subgroups of IgG4-RD patients were listed in **Table 7**. Among them, SLPI was found upregulated in Mikulicz group, Head and neck group and Retroperitoneal aorta group compared with HC. Functional enrichment analysis in plasma shows that “platelet degranulation” term was identified in all those four comparisons (**Figures S1A–D**). FGG, FGB, FGA, F8, CFD, ITIH4, QSOX1, CLU, SPP2, F5, TAGLN2, SRGN, and THBS1 were the key molecules involved in platelet degranulation related process. Interestingly, FERMT3, a gene related to platelet degranulation, was decreased in the saliva samples of Hepatobiliary group versus HC and Head and neck group versus HCs, and in the plasma samples of IgG4-RD in our previously published article. DEP identification was also performed in the plasma of IgG4-RD patients with salivary gland involvement and without salivary gland involvement. However, there were no common differentially expressed proteins were obtained in other validation datasets.

**Table 7:** Common up and down DEPs in saliva and plasma between IgG4-RD patients of four distinctive phenotypes and HC samples with same expression tendencies.

Saliva					Plasma			
A/HC	Protein	Ratio	p-value	Up/down expressed	Protein	Ratio	p-value	Up/down expressed
	SLPI	1.97	0.0278	up	SLPI	0.52	0.0401	up
	PIGR	1.32	0.0311	up	PIGR	0.79	0.0099	up
	IGKV2-24	1.28	0.0431	up	IGKV2-24	0.88	0.0232	up
	IGLV7-46	1.26	0.0264	up	IGLV7-46	1.01	0.003	up
	CLU	0.94	0.0364	up	CLU	0.43	0.0214	up
	YWHAE	-0.58	0.003	down	YWHAE	-0.51	0.0277	down
B/HC	APOB	1.98	0.0024	up	APOB	1.52	0.0125	up
	IGHG4	1.87	0.0256	up	IGHG4	2.5	0.0018	up
	IGHV1-2	1.74	0.0225	up	IGHV1-2	1.96	0.0031	up
	IGLV3-19	1.45	0.0351	up	IGLV3-19	1.96	0.0068	up
	GPX3	1.24	0.0206	up	GPX3	0.84	0.0199	up
	VCP	-0.69	0.0417	down	VCP	-0.69	0.0417	down
C/HC	LYZ	3.22	0.0063	up	LYZ	1.12	0.0004	up
	SLPI	2.93	0.0243	up	SLPI	0.81	0.0026	up
	ASL	1.53	0.0221	up	ASL	0.3	0.0341	up
	VTN	1.47	0.0254	up	VTN	0.6	0.0191	up
	APOB	1.32	0.0145	up	APOB	1.69	0.0013	up
	CAPZA2	1.32	0.0316	up	CAPZA2	0.36	0.0161	up
	GPX3	1.26	0.0037	up	GPX3	0.72	0.0048	up
	AMBP	1.05	0.016	up	AMBP	0.52	0.0155	up
	HPX	0.94	0.0174	up	HPX	0.58	0.0377	up
	TPM3	-0.95	0.0272	down	TPM3	-1.42	0.0004	down
D/HC	SLPI	2.42	0.0059	up	SLPI	0.83	0.008	up
	LYZ	1.46	0.0052	up	EHD1	-0.71	0.0426	down
	EHD1	1.15	0.0389	down	LYZ	0.89	0.0389	up

A. Mikulicz group; B. Hepatobiliary group; C. Head and neck group; D. Retroperitoneal aorta group.

## Discussion

At present, the high heterogeneity of IgG4-RD presents a considerable challenge in etiological understanding, in part because of their heterogeneous manifestations. Despite progresses in the diagnosis and treatment of IgG4-RD, the exact pathogenic mechanism of IgG4-RD with four distinctive phenotypes remains unclear (30). Several studies reported that four distinct phenotypes including pancreato-hepato-biliary disease, retroperitoneal fibrosis and/or aortitis, head and neck-limited disease, and classic Mikulicz’s syndrome with systemic involvement were a framework for further identifying IgG4-RD (8). However, the key molecular elements in these subgroups remain fully elucidated. Considering the saliva gland is one of the most involved sites of IgG4-RD patients and saliva contains serum components, lesion exudate, exfoliated cells, and proteins, in this study, we identified several differentially expressed proteins in saliva samples from IgG4-RD patients and HCs. In addition, we mainly focused on DEPs and performed functional enrichment among IgG4-RD patients with four distinct phenotypes.

In this paper, most saliva DEPs in IgG4-RD compared with HC belong to the antimicrobials category (**Figure 1B** and **Table S1**). As we know, one of the major factors responsible for the ecological equilibrium in the mouth is saliva, which in several ways affects the colonization and growth of bacteria (31). At the same time, saliva harbors a large panel of antimicrobial proteins which directly and indirectly inhibit uncontrolled outgrowth of bacteria. This article intuitively shows that there were a lot of antibacterial proteins in saliva. Indeed, we found differentially expressed proteins identified in the comparisons between each IgG4-RD subgroup and HC samples mainly belong to antimicrobials category by using IMMPORT database (**Figure S2**). We carefully checked the DEPs that were involved in antimicrobials term in each comparison and found S100 family such as S100A8, S100A9, S100A11, and S100A12 were common in this term. Till to now, several S100 proteins have been shown to play central roles in the innate immune response to infection by pathogenic organisms and have numerous associations with inflammation (32).. Moreover, altered expression of S100 proteins also correlates with multiple autoimmune disorders including systemic lupus erythematosus, Still’s, and Sjogren’s (33). As an IgG4 related disease, various immune-mediated mechanisms have been suggested that could initiate the inflammatory response (30). Some potential initiating mechanisms include bacterial infection and molecular mimicry in the setting of genetic risk factors and autoimmunity (34). This may suggest that infection may play an inducing or promoting role in the pathogenesis of IgG4-RD.

We found that “Neutrophil mediated process” related GO BP term that includes neutrophil degranulation (GO:0043312), neutrophil activation involved in immune response (GO:0002283), neutrophil activation (GO:0042119), and neutrophil mediated immunity (GO:0002446), was identified in the comparisons of Mikulicz group with Head and neck group (A vs. C), and Head and neck group with Retroperitoneal aorta group (C vs. D) (**Figures 3A, B**), which both include FCN1, MPO and HK3 (**Figures 3C, D**) and all these proteins were increased in Head and neck group compared with Mikulicz group or Retroperitoneal aorta group (**Table S2**). FCN1 is thought to function locally in inflamed lesions following secretion from monocytes, macrophages, and granulocytes, suggesting that it may play a role in systemic immunity (35). Recent reports have described the involvement of FCN1 in autoimmune diseases such as rheumatoid arthritis and FCN1 level might reflect the degree of inflammation (36). Hexokinase-3 (HK3), as a member of the hexokinase family, may stimulate the presenting surface markers on monocytes/macrophages and regulate the key immune checkpoint molecules on exhaustive T cells, thus inducing anti-tumor immune responses (37).

We found three upregulated DEPs (SLPI, QSOX1 and LGALS3) and one downregulated DEP (VCP) with expression tendencies overlapped in the saliva and plasma (**Table 6**). Galectin-3 (Gal-3), also known as LGALS3, is involved in cell differentiation, inflammation, fibrogenesis, and immune modulation (38). By using String database, we found 10 proteins, including FN1, HRAS, EGFR, KRAS, SUFU, MUC1, CTNNB1, GEMIN4, CLEC7A, LGALS3BP, showed strong relationship with LGALS3 (**Figure S3**). In this study, most saliva DEPs identified in IgG4-RD compared with HCs were predominantly enriched in neutrophil mediated GO bioprocess, including neutrophil mediated immunity and neutrophil activation. “Fc gamma R-mediated phagocytosis” process was significantly enriched in Mikulicz group compared with Head and neck group, indicating a potential role in the pathogenesis of IgG4-RD. Activation of Fcg receptors (FcgR) on phagocytes can promote phagocytosis and the subsequent antigen presentation process (39). However, immune response is a complex process, and can be modulated by multiple factors.

Considering SS is another common autoimmune disease that affects salivary and lacrimal glands, thus we performed differential gene expression analysis and functional enrichment analysis using SS and HC data from GSE40568 dataset (26). As shown in **Figure S4**, although salivary gland involvement was noted in both SS and IgG4-RD, there no common pathways were found in Sjogren syndrome and IgG4-RD patients in this study. Functional enrichment analysis indicated that the “type I interferon signaling pathway” related GO BP term was enriched in SS patients. Accumulating evidence has shown that the activated type I interferon pathway played an important role in the disease pathogenesis of Sjögren’s syndrome, responsible for the activation of autoreactive immune cells and injury of salivary glands (40). These findings suggest that IgG4-RD with salivary gland involvement may have different autoimmune pathways from SS, although they both affect salivary glands.

In this paper, we compared the DEPs identified in our previously published proteomic plasma data (11) with the screened DEPs between IgG4-RD patients and HCs in this study, we found a number of DEPs were overlapped (**Table S5**). Thus, we will examine the potential of these proteins in the differential diagnosis of IgG4-RD using a larger number of IgG4-RD samples in future studies.

To our knowledge, this is the first proteomic analysis of saliva samples from four subgroups of IgG4-RD patients. There are also limitations in our study. Firstly, the sample size of our analysis was relatively small; secondly, other types of omics (e.g., lipidomics, metabolomics, and glycomics) and validation with laboratory experiments were not carried out in this study and will be investigated in future studies.

## Data availability statement

In this study, our proteomics data of saliva and plasma samples from ten IgG4-RD patients and five healthy controls (HCs) were deposited to the ProteomeXchange Consortium *via* the iProX partner repository with the dataset identifier PXD036552 (http://proteomecentral.proteomexchange.org/cgi/GetDataset?ID=PXD036552). Datasets of GSE66465 and GSE40568 were downloaded from Gene Expression Omnibus Dataset (GEO Dataset: http://www.ncbi.nlm.nih.gov/geo/).

## Ethics statement

The studies involving human participants were reviewed and approved by Tongji Hospital, Tongji Medical College, Huazhong University of Science, and Technology Institutional Review Board. The patients/participants provided their written informed consent to participate in this study.

## Author contributions

S-YL analyzed the data and wrote the manuscript. TZ made the sample collection. SC and Z-WH provided specific knowledge on IgG4-RD. JZ and LD designed the study and revised the manuscript. All authors contributed to the article and approved the submitted version.

## Funding

This study was supported by grants from the National Natural Science Foundation of China (NSFC Nos. 81771754), and Tongji Hospital Clinical Research Flagship Program (2019CR206).

## Conflict of interest

The authors declare that the research was conducted in the absence of any commercial or financial relationships that could be construed as a potential conflict of interest.

## Publisher’s note

All claims expressed in this article are solely those of the authors and do not necessarily represent those of their affiliated organizations, or those of the publisher, the editors and the reviewers. Any product that may be evaluated in this article, or claim that may be made by its manufacturer, is not guaranteed or endorsed by the publisher.

## Supplementary material

Supplementary Figure 1
**(A)** Enriched GO and KEGG terms of DEPs in comparison of Mikulicz group vs. HC in plasma. **(B)** Enriched GO and KEGG terms of DEPs in comparison of Hepatobiliary group vs. HC in plasma. **(C)** Enriched GO and KEGG terms of DEPs in comparison of Head and neck group vs. HC in plasma. **(D)** Enriched GO and KEGG terms of DEPs in comparison of Retroperitoneal aorta group vs. HC in plasma.

Supplementary Figure 2 Immune categories of DEPs identified in saliva between each IgG4-RD subgroup and HC samples. Different color represents various immune related categories from IMMPORT database. **(A)** Mikulicz group; **(B)** Hepatobiliary group; **(C)** Head and neck group; and **(D)** Retroperitoneal aorta group.

Supplementary Figure 3 Protein interactive network of LGALS3.

Supplementary Figure 4
**(A)** Enriched GO and KEGG terms of differentially expressed genes in comparison of Sjögren’s syndrome vs. HCs in labial salivary glands (LSGs). **(B)** Top 20 Enriched GO terms of differentially expressed genes in comparison of Sjögren’s syndrome vs. HCs in labial salivary glands (LSGs).

Supplementary Table 1 The immune categories of DEPs identified in IgG4-RD and HC in salivary by using IMMPORT database.

Supplementary Table 2 DEPs identified in the comparisons between IgG4-RD with four subgroups.

Supplementary Table 3 DEPs identified in the comparisons between IgG4-RD with four subgroups and HC.

Supplementary Table 4 DEPs identified in the comparisons between IgG4-RD and HC.

Supplementary Table 5 Overlapped up DEPs in IgG4-RD patients in plasma in two datasets.
