# Supplementary figures and images for: Proteomic characteristics of saliva in patients with different subgroups of IgG4-RD
Source: Front Immunol. 2022 Nov 22;13:1026921. doi: 10.3389/fimmu.2022.1026921 (PMC9723444; doi:10.3389/fimmu.2022.1026921)

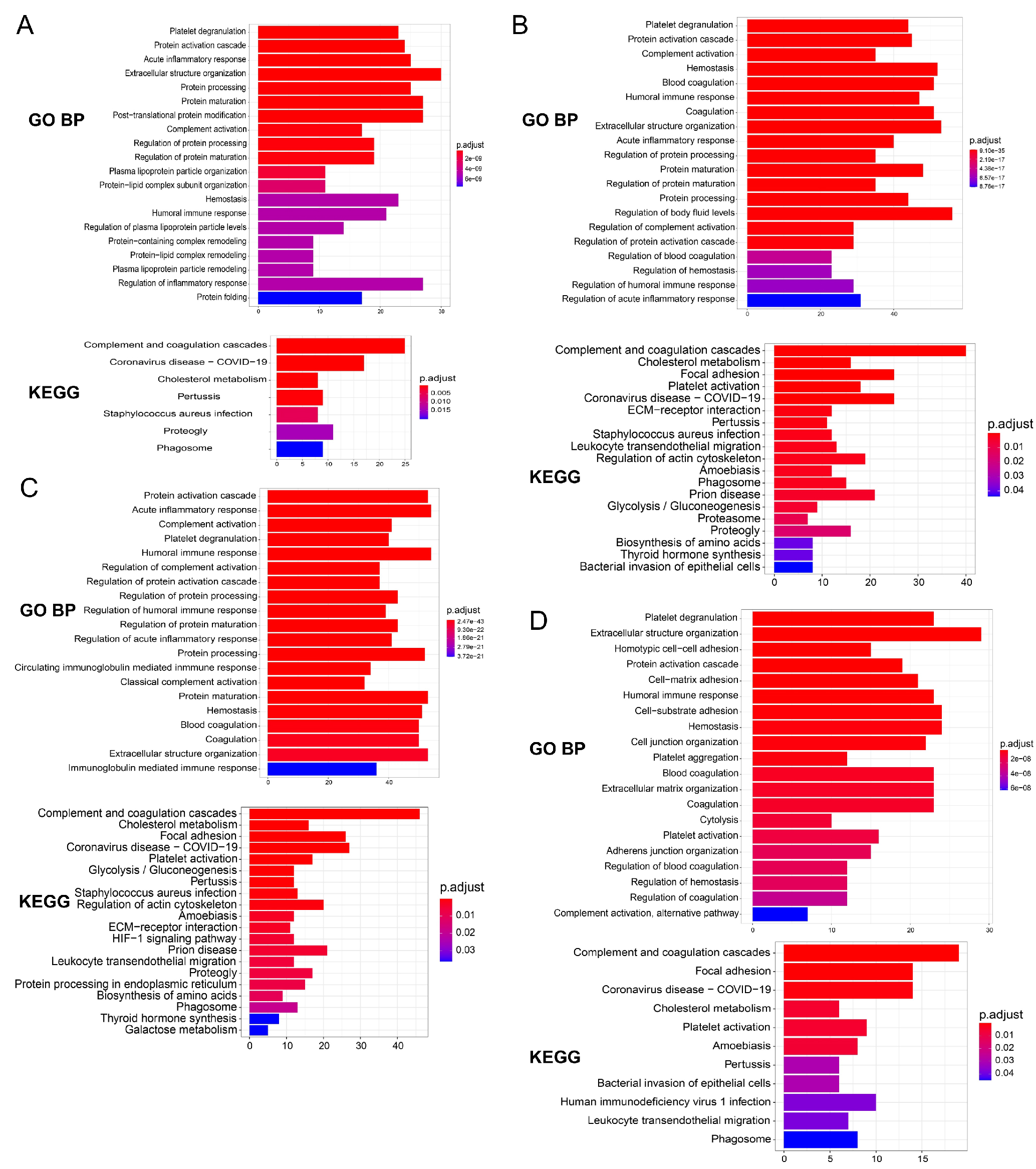

Supplement: Supplementary Figure 1 — (A) Enriched GO and KEGG terms of DEPs in comparison of Mikulicz group vs. HC in plasma. (B) Enriched GO and KEGG terms of DEPs in comparison of Hepatobiliary group vs. HC in plasma. (C) Enriched GO and KEGG terms of DEPs in comparison of Head and neck group vs. HC in plasma. (D) Enriched GO and KEGG terms of DEPs in comparison of Retroperitoneal aorta group vs. HC in plasma. [file Image_1.jpeg]

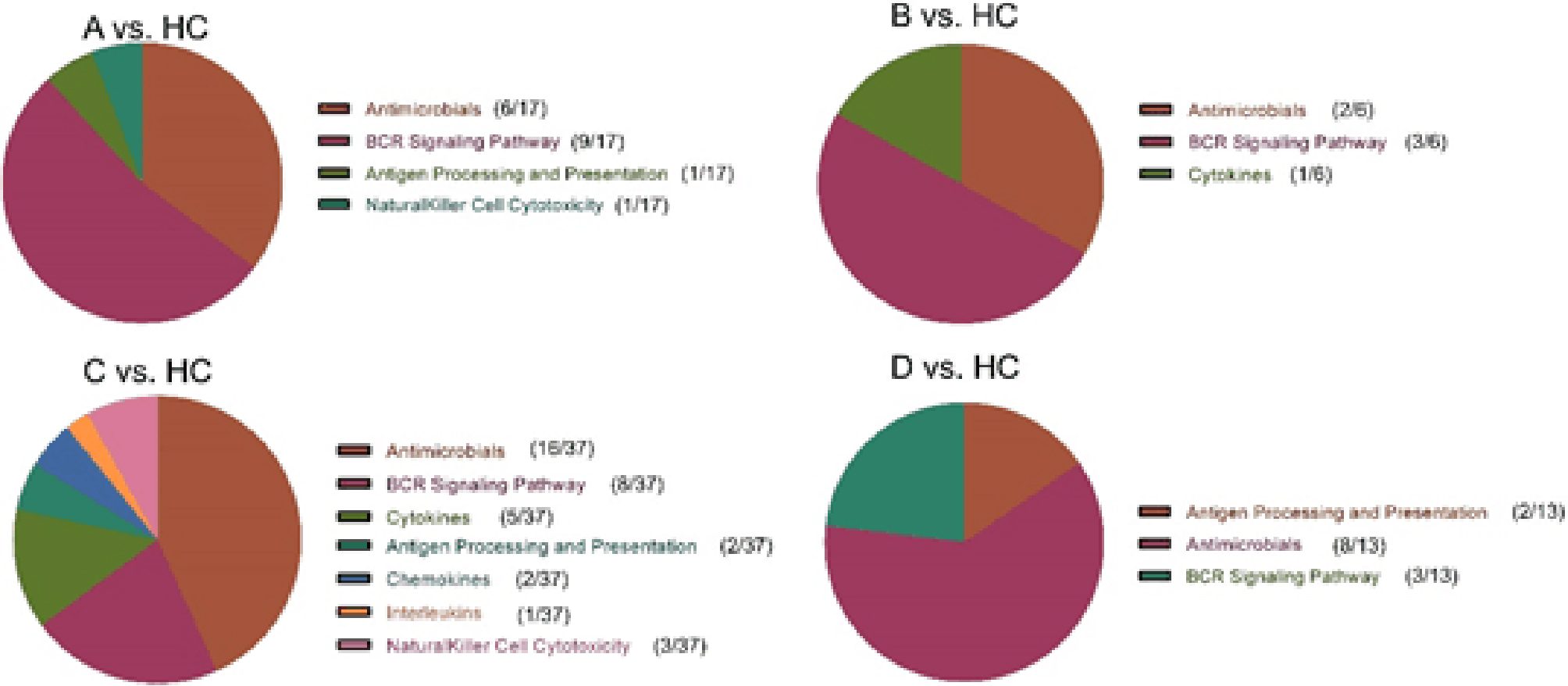

Supplement: Supplementary Figure 2 — Immune categories of DEPs identified in saliva between each IgG4-RD subgroup and HC samples. Different color represents various immune related categories from IMMPORT database. (A) Mikulicz group; (B) Hepatobiliary group; (C) Head and neck group; and (D) Retroperitoneal aorta group. [file Image_2.jpeg]

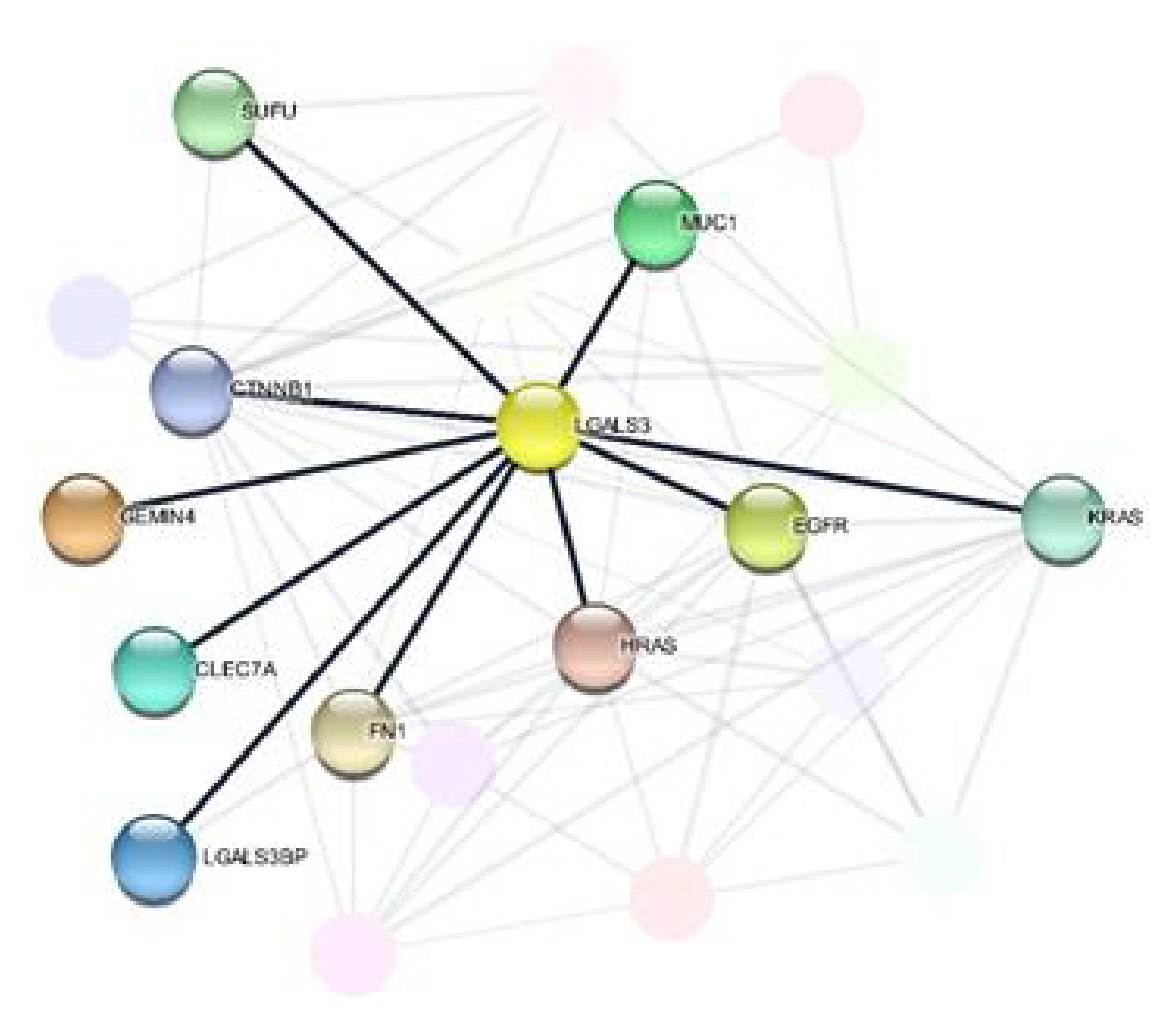

Supplement: Supplementary Figure 3 — Protein interactive network of LGALS3. [file Image_3.jpeg]

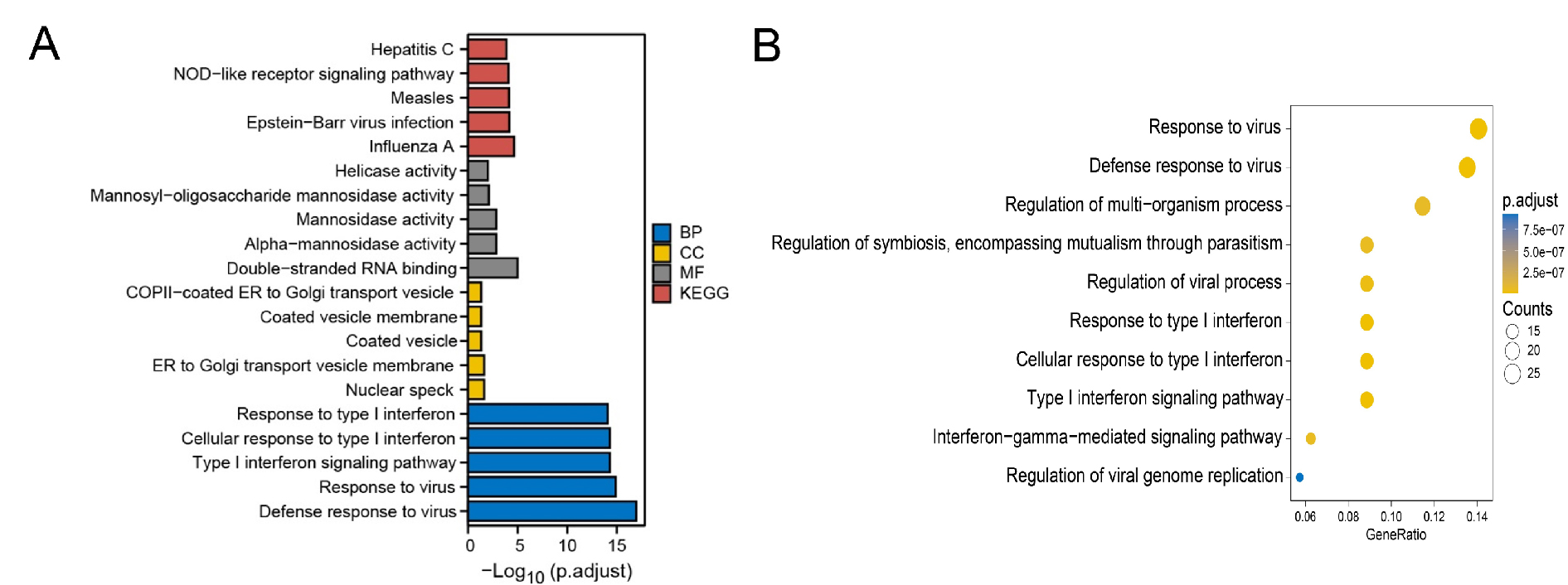

Supplement: Supplementary Figure 4 — (A) Enriched GO and KEGG terms of differentially expressed genes in comparison of Sjögren’s syndrome vs. HCs in labial salivary glands (LSGs). (B) Top 20 Enriched GO terms of differentially expressed genes in comparison of Sjögren’s syndrome vs. HCs in labial salivary glands (LSGs). [file Image_4.jpeg]
